# Disseminated intravascular coagulation as a prognostic factor in heatstroke: a retrospective cohort study based on Japanese insurance claims

**DOI:** 10.3389/fmed.2026.1941386

**Published:** 2026-09-04

**Authors:** Hiroyuki Koami, Yutaro Furukawa, Hiroki Ushijima, Daisuke Muroya, Tadashi Matsuoka, Takaaki Totoki, Kenji Kandori, Takahiro Oishi, Kasumi Satoh, Yutaka Umemura, Kazuma Yamakawa, Mineji Hayakawa, Kohji Okamoto, Yuichiro Sakamoto

**Affiliations:** 1 Department of Emergency and Critical Care Medicine, Saga University, Saga, Japan; 2 LOCOMOCO (Landmark of Clinical Observations in MicrOcirculation and Coagulation Outcomes) Study Group, Tokyo, Japan; 3 Department of Emergency Medicine, Kitakyushu City Yahata Hospital, Kitakyushu, Fukuoka, Japan; 4 Department of Emergency and Critical Care Medicine, Keio University, Tokyo, Japan; 5 Department of Emergency and Critical Care Medicine, Osaka Medical and Pharmaceutical University, Takatsuki, Osaka, Japan; 6 Department of Emergency and Critical Care Medicine, Japanese Red Cross Society Kyoto Daini Hospital, Kyoto, Japan; 7 Department of Emergency and Critical Care Medicine, Kansai Medical University, Osaka, Japan; 8 Department of Emergency and Critical Care Medicine, Akita University Graduate School of Medicine, Akita, Japan; 9 Department of Traumatology and Acute Critical Medicine, Osaka University Graduate School of Medicine, Suita, Japan; 10 Department Emergency and General Medicine, Sapporo City General Hospital, Sapporo, Japan; 11 Department of Surgery, Kitakyushu City Yahata Hospital, Kitakyushu, Fukuoka, Japan

**Keywords:** disseminated intravascular coagulation, heatstroke, JAAM-2 DIC criteria, mortality, multiple organ failure, nationwide database

## Abstract

**Purpose:**

Heatstroke is an emerging global health concern, particularly among the elderly. Disseminated intravascular coagulation (DIC) complicates about 20% of these cases and contributes to poor outcomes. The JAAM-2 DIC score, which excludes SIRS, has been validated in sepsis. However, its utility in heatstroke, a condition sharing pathophysiological features with sepsis, remains unexamined. To evaluate the association between heatstroke-associated DIC, defined by JAAM-2 criteria, and clinical outcomes, using a nationwide Japanese database of medical claims.

**Methods:**

We conducted a retrospective analysis using data from Japanese health insurance claims from 2014 to 2023. Among 2,633 adult patients admitted with heatstroke, 606 cases with complete DIC data on admission were included. Patients were categorized into DIC (*n* = 76) and non-DIC groups (*n* = 530) based on DIC criteria. Multivariate logistic regression analysis was performed to evaluate DIC as a prognostic indicator of poor outcomes.

**Results:**

The DIC group was older and exhibited higher rates of impaired consciousness, hepatic and renal dysfunction, bleeding/thrombotic complications, and mortality. Kaplan–Meier analysis showed significantly lower 28-day survival in the DIC group (log-rank *P* < 0.0001). In the multivariate model, DIC remained associated with in-hospital mortality (odds ratio 1.566, 95% CI 1.147–2.140, *P* = 0.004). Sub-analyses showed three factors strongly related to poor prognosis: initial DIC score ≥4, FDP sub-score of 3 at presentation, and D1+/D2 + or D1-/D2 + pattern on day 2.

**Conclusion:**

JAAM-2 DIC is associated with poor clinical outcomes in patients with heatstroke, supporting the clinical importance of recognizing coagulopathy in this population.

## Introduction

Heatstroke has emerged as a major global health concern, driven in part by global warming. When physiological thermoregulatory capacity becomes overwhelmed by environmental heat stress or strenuous exercise, systemic inflammation may ensue, leading to central nervous system dysfunction, hepatic injury, renal failure, and disseminated intravascular coagulation (DIC), complications associated with multiple organ failure and poor outcomes (1, 2).

DIC has been reported in 2.6% to 28.4% of heatstroke patients and is significantly associated with in-hospital mortality (3–5). Mortality rate in heatstroke DIC patients increases in parallel with higher Japanese Association for Acute Medicine (JAAM)-DIC scores, which incorporate Systemic Inflammatory Response Syndrome (SIRS) criteria (3). However, the clinical relevance of SIRS has diminished, prompting the need for diagnostic approaches that are not dependent on SIRS-based scoring (6, 7).

JAAM-2 DIC criteria were developed to preserve diagnostic utility while aligning with the contemporary sepsis definition by removing SIRS components (8). These criteria have proven reliable for predicting poor clinical outcomes and initiating anticoagulant therapy in sepsis patients. Nevertheless, evidence regarding the clinical applicability of the DIC score in non-septic conditions such as heatstroke remains limited. Given that heatstroke-associated DIC may share several pathophysiological mechanisms with sepsis, including systemic inflammation, endothelial injury, and reduced fibrinolysis, evaluating the prognostic value of DIC in this population is clinically important (2).

To date, no study has assessed the utility of the JAAM-2 DIC score for revealing heatstroke patients at high-risk of poor clinical outcomes using a large-scale dataset. Establishing this association contributes to a more objective and practical framework for early risk stratification and informs targeted DIC management strategies.

## Materials and methods

### Data source

We utilized commercially available, anonymized, insurance claim data from the Japan Medical Data Center (JMDC Inc.; Tokyo, Japan). The JMDC database contains comprehensive healthcare data, comprising a payer database of 17 million registered members since 2005, a personal health record database covering 5.7 million members, a hospital database spanning 860 healthcare facilities, a pharmacy database incorporating 5,700 pharmacies, and an electronic medical record database including 190 contracted medical institutions, which collectively represent approximately 14% of the Japanese population as of 2024 (JMDC Database: https://www.jmdc.co.jp/en/). All medical institutions used ICD-10-based diagnostics when claiming reimbursement for medical expenses. An anonymized dataset from 2014 to 2023 was acquired from JMDC in September 2023. Data were then extracted and subjected to analysis employing necessary parameters and designated identifiers.

### Study design

**Figure 1:**
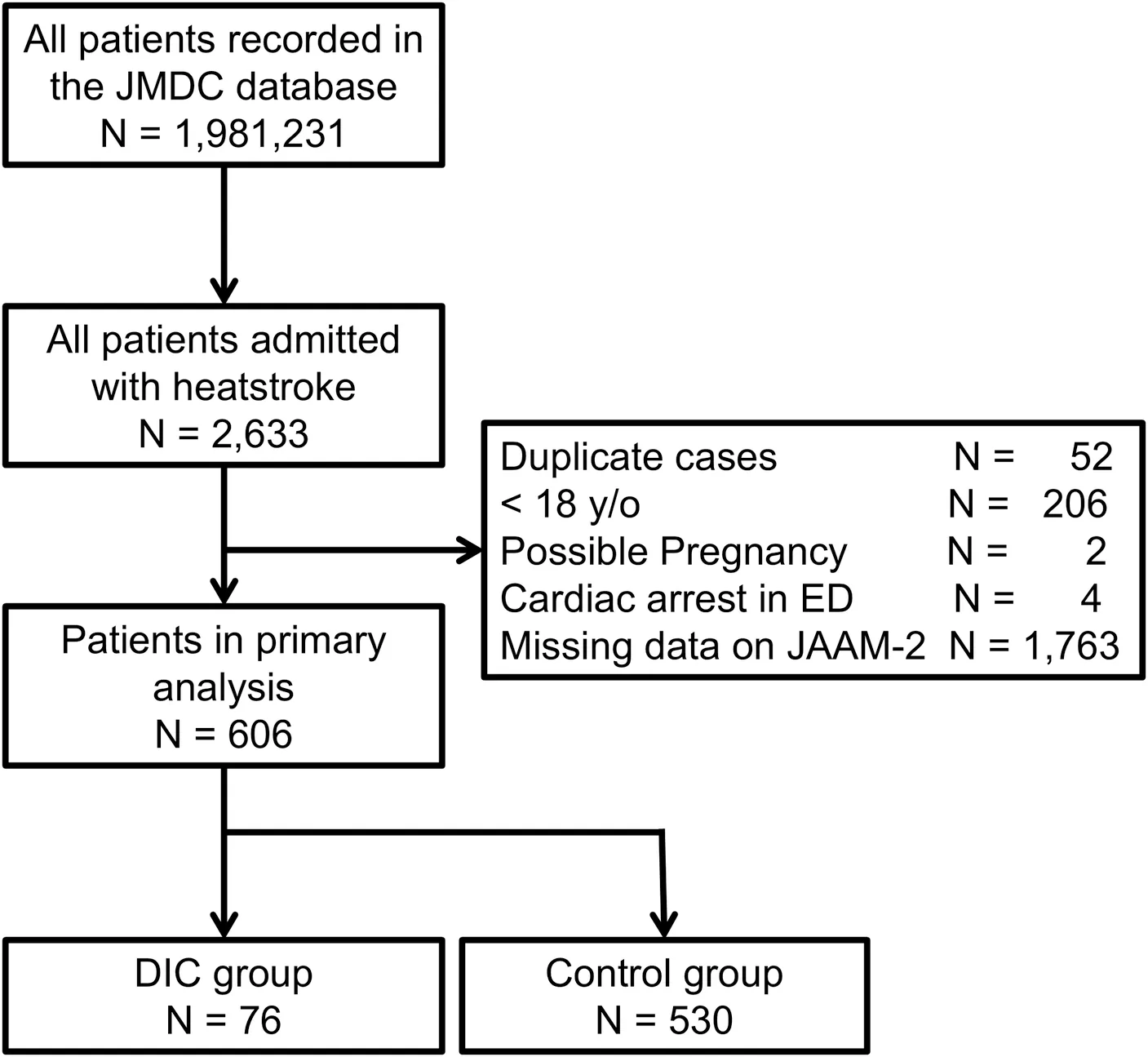
Study flow of primary analysis. ED, emergency department; JAAM-2, modified version of Japanese association for acute medicine; DIC, disseminated intravascular coagulation.

This retrospective study included all hospitalized patients in the JMDC database diagnosed with heatstroke (Figure 1). We only included data from the earliest hospitalization for patients who had been hospitalized multiple times. Patients under 18 years old, with possible pregnancy, cardiac arrest in the emergency department, or missing DIC scores on the day of admission were excluded. For the primary analysis, all remaining patients were divided into two groups based on the presence or absence of DIC on the first day of heatstroke: the DIC group and the control group.

For secondary analysis, only patients who underwent DIC measurements on both the first and second days of illness were extracted (Supplementary Figure 1). All patients identified were divided into four groups based on whether they were diagnosed with DIC on the first or second day of heatstroke: D1+/D2 + group [Day1(+)/Day2(+)], D1+/D2- group [Day1(+)/Day2(-)], D1-/D2 + group [Day1(-)/Day2(+)], and D1-/D2- group [Day1(-)/Day2(-)].

This study followed conduct and reporting guidelines of the STROBE (Strengthening the Reporting of Observational Studies in Epidemiology) statement (9). The present study, which involved human participants was approved by Osaka Medical and Pharmaceutical University (approval number: 2023-112-1). All procedures were performed in accordance with local legislation and institutional requirements. The ethics committee or institutional review board waived the requirement for written informed consent from participants, their legal guardians, or next of kin due to the retrospective design of the study and the use of anonymized data.

### Primary/ secondary outcomes

The primary outcome was in-hospital mortality. Secondary outcomes included 28-day mortality, length of hospital stay, bleeding complications, deep vein thrombosis and (DVT)/pulmonary embolism (PE). In-hospital mortality was defined as death from any cause during hospitalization. Bleeding complications and DVT/PE were identified based on ICD-10 codes (Supplementary Table 1).

### Data collection

In the present study, the following variables were collected and analyzed: patient demographics [age, sex, transportation by ambulance, modified Charlson index (10)], clinical severity indices related to heatstroke (proportion of third-degree heatstroke (3), Japan coma scale (JCS) on admission (11), JAAM-2 DIC score (8), International Society on Thrombosis and Haemostasis (ISTH) overt DIC score (12), ISTH overt DIC diagnosis), blood findings (platelet count, prothrombin time-international normalized ratio (PT-INR), activated partial thromboplastin time (aPTT), fibrinogen, fibrin/fibrinogen degradation products (FDP), D-dimer, antithrombin (AT) activity, creatine kinase (CK), total bilirubin (T-BiL), creatinine (Cre), C-reactive protein (CRP), lactate), treatment modalities [mechanical ventilation, renal replacement therapy, use of vasopressor, AT therapy, and recombinant thrombomodulin (rTM) therapy, heparin use, and transfusion].

### Definitions

In this study, we defined heatstroke as a condition with an ICD-10 code of T67.0–T67.9 (Supplementary Table 1). The definition of severe grade of heatstroke was also defined by JAAM heatstroke (JAAM-HS) criteria, which included ICD-10 of T67.0 with any of the following conditions: central nervous system manifestations, hepatic/renal dysfunction required in-hospital care, or DIC (Supplementary Table 2) (13). JCS classifications are also shown in Supplementary Table 2. This study employed a broad classification (0, 1-digit, 2-digit, 3-digit) to assess the general nature of impaired consciousness. Furthermore, the JAAM-2 score for newly diagnosed DIC, as well as ISTH-overt DIC scores, are summarized in Supplementary Table 3. A diagnosis of DIC with the JAAM-2 score requires a total ≥3, and diagnoses of ISTH-overt DIC were defined as scores of ≥5. Due to inter-assay variability in D-dimer measurements, FDP values were used whenever possible. When FDP values were unavailable, D-dimer values were used to calculate the fibrin-related marker component of the JAAM-2 DIC score. Furthermore, because the specific measurement kits for D-dimer at each institution could not be identified in the database, we defined the cut-off values as 6 μg/mL for 1 point and 15 μg/mL for 3 points, based on a previous validated cohort study utilizing the same JMDC database (14).

### Statistical analysis

Continuous variables are presented as medians with interquartile ranges [Median (Q1-Q3)], while nominal variables are shown as numbers with percentages [*N* (%)]. The Wilcoxon test was employed to analyze continuous variables, and the Chi-square test and Fisher's exact tests were employed for categorical/nominal variables. A *P* value less than 0.05 was considered statistically significant for all analyses. We performed primarily univariate analyses to evaluate the association between heatstroke-associated DIC and each variable (patient characteristics, clinical severity scores, laboratory data, treatment profiles, complications during hospitalization, and clinical outcomes). Kaplan–Meier curves were plotted, and log-rank tests conducted to assess 28-day survival rates in relation to the presence or absence of heatstroke-associated DIC. Logistic regression analyses were performed to determine whether DIC derived from heatstroke was an independent factor associated with in-hospital mortality. Adjustment factors were determined based on previous reports on heatstroke and Japanese guidelines (15). Briefly, the following variables were included in multivariable models: age (continuous), JCS (categorical, using the standard 0/1-digit/2-digit/3-digit classification), creatinine (continuous), T-Bil (continuous), and JAAM-2 DIC score (continuous). JCS was entered as a categorical variable because its ordinal structure does not represent a linear gradient suitable for continuous modeling. Additionally, JAAM-2 DIC score was entered as a continuous variable, following its established linear association with disease severity in prior studies (8). Next, odds ratios (OR) and their corresponding 95% confidence intervals (CI) were calculated to assess the association between various factors and in-hospital mortality after heatstroke. For DIC criteria, platelet count, PT and FDP sub-scores, the “Negative” and “0” categories, respectively, served as reference groups (OR=1). Finally, Kaplan–Meier curves were created, and log-rank tests were used to evaluate the presence or absence of DIC diagnosis on days 1 and 2 after heatstroke, as well as 28-day survival. All statistical analyses utilized JMP® Pro 16.1.0 software (SAS Institute, Cary, NC, USA).

## Results

A total of 2,633 heatstroke patients were extracted from the original JMDC database (Figure 1). Of these cases, 606 were retained after excluding 2,027 cases that met the exclusion criteria. Included patients were divided into two groups based on the diagnosis of DIC on day 1: the DIC group (*N* = 76) and the control group (*N* = 530).

Baseline patient characteristics are summarized in Table 1. Patients in the DIC group were older [DIC group; 82 y/o [74–88] vs. Control group; 77 y/o [67–85]] and had higher modified Charlson index scores. Sex distribution and ambulance transport did not differ between groups. According to the clinical severity score, the DIC group had a higher incidence of third-degree heatstroke and severe consciousness impairment, higher JAAM-2 scores [3 [3–4] vs. 0 [0–1]] and ISTH-overt DIC scores [3 [3–3] vs. 0 [0–0]], and a higher rate of ISTH-overt DIC on day 1.

**Table 1:** Baseline patient characteristics according to DIC status in heatstroke patients.

Characteristic	JAAM-2 DIC Group			Control Group		
Age, y/o	82	[74–88]	(*N* = 76)	77	[67–85]	(*N* = 530)
Male, N (%)	45	(59.2)	(*N* = 76)	336	(63.4)	(*N* = 530)
Transport by ambulance, N (%)	68	(89.5)	(*N* = 76)	446	(84.2)	(*N* = 530)
Modified Charlson index, pts	6	[5–8]	(*N* = 76)	5	[4–7]	(*N* = 530)
JAAM-HS, severe, N (%)	56	(73.7)	(*N* = 76)	232	(43.8)	(*N* = 530)
JCS on admission, N (%)			(*N* = 76)			(*N* = 530)
0	32	(42.1)		330	(62.3)	
1–3	23	(30.3)		138	(26.0)	
10–30	9	(11.8)		34	(6.4)	
100–300	12	(15.8)		28	(5.3)	
JAAM-2 DIC score on day 1, pts	3	[3–4]	(*N* = 76)	0	[0–1]	(*N* = 530)
ISTH-overt DIC score on day 1, pts	3	[3–3]	(*N* = 36)	0	[0–0]	(*N* = 247)
Positive for overt DIC on day 1, N (%)	2	(5.6)	(*N* = 36)	0	(0.0)	(*N* = 247)

Median [Q1, Q3] and number (N) (%), *P* value <0.05. For continuous variables that were significantly different by the Wilcoxon test was performed. For nominal variables, Chi-square test and Fisher's exact tests were performed.

JAAM-2, modified version of Japanese association for acute medicine; DIC, disseminated intravascular coagulation; JAAM-HS, JAAM-heatstroke criteria; JCS, Japan coma scale; 0, fully awake; 1–3*,* awake without stimulation; 10–30, arousable with stimulation; 100–300, unarousable; ISTH, international society on thrombosis and haemostasis; PT-INR, prothrombin time-international normalized ratio; aPTT, activated partial thromboplastin time; FDP, fibrin/fibrinogen degradation products; AT, antithrombin; CK, creatine kinase; T-BiL, total bilirubin; Cre, creatinine; CRP, C-reactive protein; rTM, recombinant thrombomodulin; DVT/PE, deep vein thrombosis/ pulmonary embolism; Hp, hospital.

**Figure 2:**
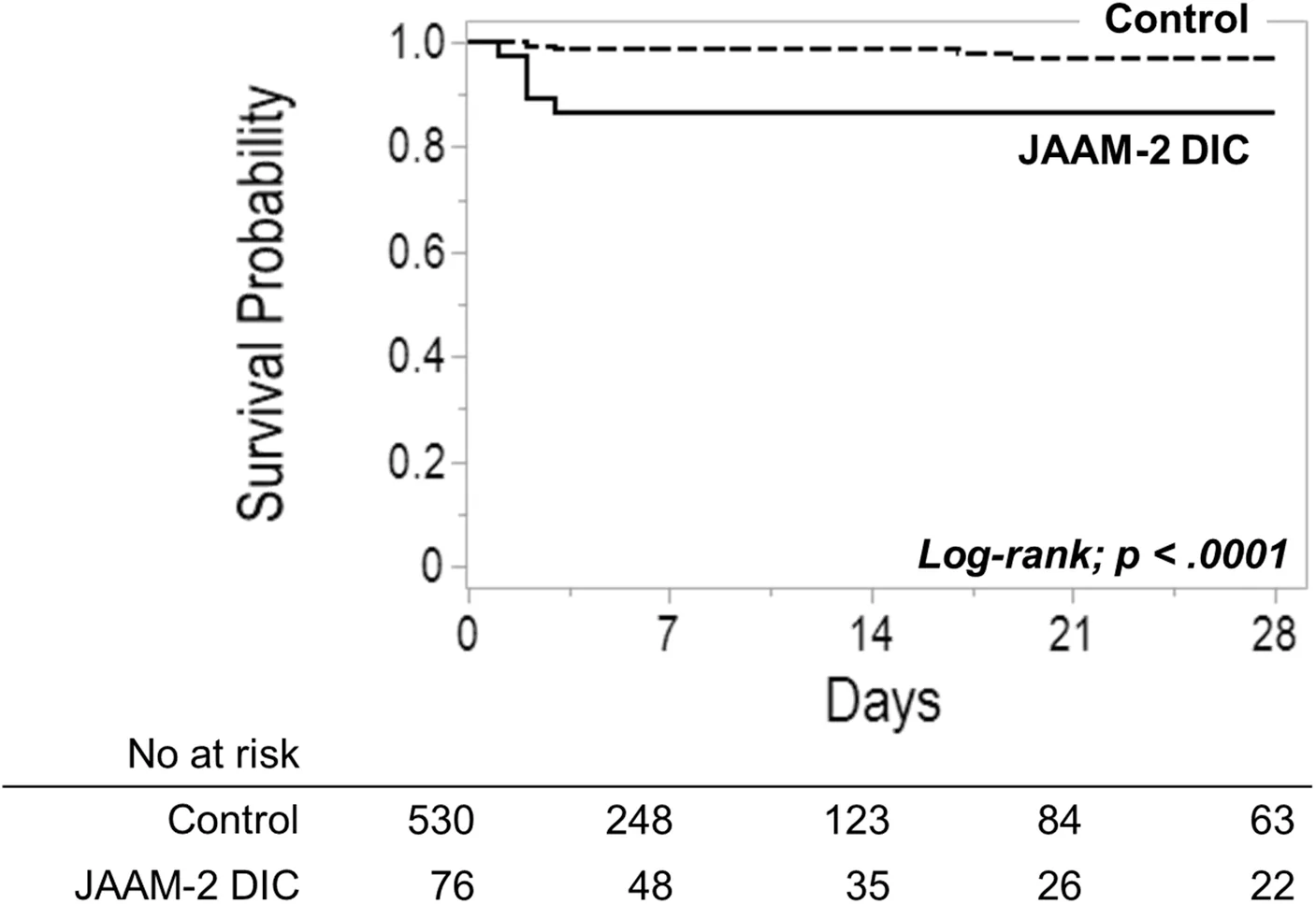
Survival analysis of JAAM-2 DIC and 28-day survival. The 28-day survival rate for DIC due to heatstroke was significantly worse compared to non-DIC cases, using the log-rank test with Kaplan–Meier curves. JAAM-2, modified version of Japanese association for acute medicine; DIC, disseminated intravascular coagulation.

Table 2 demonstrates laboratory data, treatment modalities, and clinical outcomes among the groups. The DIC group showed significantly lower platelet counts, prolonged PT-INR and APTT, and significantly higher FDP and D-dimer levels. Similarly, the DIC group showed significant increases in CK, T-BiL [1.2 mg/dL [0.7–1.7] vs. 0.8 mg/dL [0.6–1.2], *P* = 0.0001], and Cre [1.4 mg/dL [1.1–2.2] vs. 1.1 mg/dL [0.8–1.7], *P* < 0.0001]. Treatments such as mechanical ventilation, vasopressors, rTM, heparin, and blood transfusion were more frequently used for the DIC group. Bleeding complications and DVT/PE were significantly more frequent in the DIC group. The mortality rate during the first 28 days (13.2% vs. 1.3%, *P* < 0.0001) and all-cause in-hospital mortality were significantly higher in the DIC group, and the length of hospital stay was also significantly longer. Kaplan–Meier curves for 28-day survival rates with and without DIC after heatstroke showed that DIC patients with heatstroke had significantly poorer 28-day survival rates (Log-rank *P* < 0.0001) (Figure 2).

**Table 2:** Laboratory data, treatment modalities, and outcomes according to DIC status in heatstroke patients.

Characteristic	JAAM-2 DIC Group			Control Group			*P* value
Platelet, 10^4^/µL	15.6	[10.4–19.9]	(*N* = 76)	20.5	[16.2–25.6]	(*N* = 530)	<0.0001
PT-INR	1.15	[1.05–1.34]	(*N* = 47)	1.04	[0.97–1.14]	(*N* = 305)	<0.0001
aPTT, second	29	[26–35]	(*N* = 65)	28	[25–31]	(*N* = 483)	.026
Fibrinogen, mg/dL	292	[236–417]	(*N* = 36)	316	[265–388]	(*N* = 247)	.327
FDP, µg/mL	37.4	[27.5–80.7]	(*N* = 47)	5.3	[2.9–9.1]	(*N* = 191)	<0.0001
D-dimer, µg/mL	19.9	[13.5–41.7]	(*N* = 60)	1.5	[0.7–3.4]	(*N* = 457)	<0.0001
AT activity, %	84	[74–91]	(*N* = 5)	100	[87–115]	(*N* = 26)	.044
CK, U/L	325	[82–1178]	(*N* = 75)	149	[82–349]	(*N* = 525)	.002
T-BiL, mg/dL	1.2	[0.7–1.7]	(*N* = 74)	0.8	[0.6–1.2]	(*N* = 513)	.0001
Cre, mg/dL	1.4	[1.1–2.2]	(*N* = 76)	1.1	[0.8–1.7]	(*N* = 529)	<0.0001
CRP, mg/dL	0.4	[0.1–3.1]	(*N* = 70)	0.3	[0.1–1.4]	(*N* = 480)	.255
Lactate, mg/dL	21.4	[16.7–31.9]	(*N* = 8)	20.0	[14.7–35.2]	(*N* = 63)	.750
Mechanical ventilation, N (%)	9	(11.8)	(*N* = 76)	7	(1.3)	(*N* = 530)	<0.0001
Renal replacement therapy, N (%)	1	(1.3)	(*N* = 76)	3	(0.6)	(*N* = 530)	.416
Vasopressor, N (%)	12	(15.8)	(*N* = 76)	12	(2.3)	(*N* = 530)	<0.0001
AT, N (%)	1	(1.3)	(*N* = 76)	1	(0.2)	(*N* = 530)	.109
rTM, N (%)	3	(4.0)	(*N* = 76)	3	(0.6)	(*N* = 530)	.005
Heparin, N (%)	14	(18.4)	(*N* = 76)	26	(4.9)	(*N* = 530)	<0.0001
Transfusion, N (%)	5	(6.6)	(*N* = 76)	11	(2.1)	(*N* = 530)	.022
Bleeding, N (%)	5	(6.6)	(*N* = 76)	8	(1.5)	(*N* = 530)	.004
DVT/PE, N (%)	6	(7.9)	(*N* = 76)	5	(0.9)	(*N* = 530)	<0.0001
28-day mortality, N (%)	10	(13.2)	(*N* = 76)	7	(1.3)	(*N* = 530)	<0.0001
In-Hp mortality_all cause, N (%)	10	(13.2)	(*N* = 76)	10	(1.9)	(*N* = 530)	<0.0001
Length of Hp stay, days	10	[4–30]	(*N* = 76)	6	[3–13]	(*N* = 530)	.003

Median [Q1, Q3] and number (N) (%), *P* value <0.05. For continuous variables that were significantly different by the Wilcoxon test was performed. For nominal variables, Chi-square test and Fisher's exact tests were performed.

JAAM-2, modified version of Japanese association for acute medicine; DIC, disseminated intravascular coagulation, PT-INR, prothrombin time-international normalized ratio; aPTT, activated partial thromboplastin time; FDP, fibrin/fibrinogen degradation products; AT, antithrombin; CK, creatine kinase; T-BiL, total bilirubin; Cre, creatinine; CRP, C-reactive protein; rTM, recombinant thrombomodulin; DVT/PE, deep vein thrombosis/ pulmonary embolism; Hp, hospital.

**Figure 3:**
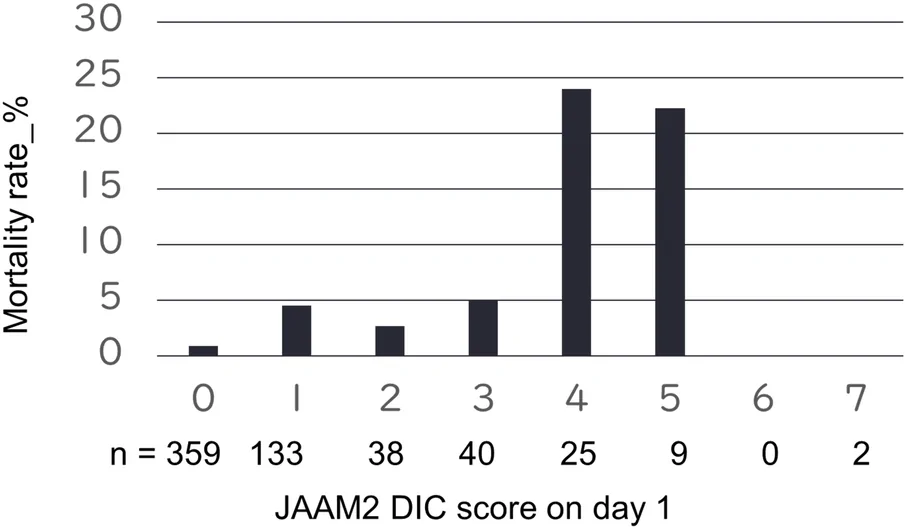
JAAM-2 DIC score and mortality rate. Although the number of most severe cases with JAAM-2 DIC scores of 6 or 7 was limited, mortality rates clearly increased in cases with JAAM-2 DIC scores of 4 or higher. JAAM-2, modified version of Japanese association for acute medicine; DIC, disseminated intravascular coagulation.

Next, we examined the correlation between DIC score and mortality rate (Figure 3). Although the number of people with JAAM-2 DIC scores of 6 or 7 is extremely limited, higher JAAM-2 DIC scores were associated with higher in-hospital mortality, particularly among patients with scores ≥4.

We then developed a logistic regression model (Table 3). This model used factors identified from previous publications to assess whether an increase in DIC score reliably detects in-hospital mortality in patients with heatstroke. Higher DIC score emerged as a significant predictor of in-hospital mortality (OR: 1.566, 95%CI: 1.147–2.140, *P* = 0.004). In a sensitivity analysis incorporating the modified Charlson Index, the association between the JAAM-2 DIC score and in-hospital mortality remained essentially unchanged (OR, 1.633; 95% CI, 1.202–2.227), and remained similar when both age and modified Charlson index were included in the model (OR, 1.595; 95% CI, 1.166–2.183) (Supplementary Table 4).

**Table 3:** Multivariate logistic regression model for in-hospital mortality in heatstroke patients.

Characteristic	Univariate analysis			Multivariate analysis		
	OR	95%CI	*P* value	OR	95%CI	*P* value
Age	1.026	0.995–1.063	.137	1.040	0.998–1.092	.095
JCS [3-/2-digit]	7.776	1.907–52.736	.011	13.350	2.158–82.595	.005
Cre	1.208	0.875–1.534	.170	1.196	0.778–1.670	.343
T-BiL	1.012	0.512–1.565	.967	0.747	0.333–1.426	.434
JAAM-2 DIC score	1.838	1.440–2.360	< 0.0001	1.566	1.147–2.140	.004

*P* value <0.05. Regarding JCS, it showed the odds ratio for in-hospital mortality in cases of impaired consciousness with 3-digit scores compared to those with 2-digit scores.

OR, odds ratio; 95%CI, 95% confidence interval; JCS, Japan coma scale; 0, fully awake; 1–3(1-digit), awake without stimulation; 10–30(2-digit), arousable with stimulation; 100–300(3-digit), unarousable; Cre, creatinine; T-BiL, total bilirubin; JAAM-2, modified version of Japanese association for acute medicine; DIC, disseminated intravascular coagulation.

Table 4 presents odds ratios of DIC criteria and their sub-scores for in-hospital mortality after heatstroke. Positive DIC criteria were significantly associated with an increased risk of in-hospital mortality (OR: 7.88, 95% CI: 3.12–19.90, *P* < 0.0001). A PT sub-score of 1 also demonstrated a significant association (OR: 3.80, 95% CI: 1.53–9.47, *P* = 0.005). FDP sub-scores of 1 (OR: 4.52, 95% CI: 1.28–15.33, *P* = 0.021) or 3 (OR: 12.74, 95% CI: 4.42–39.33, *P* < 0.0001) showed a significant association with in-hospital mortality. In contrast, platelet sub-scores were not significantly associated with mortality.

**Table 4:** Odds ratios of JAAM-2 and sub-scores of each component for in-hospital mortality after heatstroke.

Characteristic	No (%)	Mortality	OR	95%CI	*P* value
JAAM-2 DIC criteria					
Negative	530 (87.5)	1.9	1 [reference]	-	-
Positive	76 (12.5)	13.2	7.88	(3.12–19.90)	< 0.0001
Platelet sub-score					
0	546 (90.1)	2.9	1 [reference]	-	-
1	49 (8.1)	6.1	2.16	(0.49–6.79)	.273
3	11 (1.8)	9.1	3.31	(0.18–18.94)	.336
PT-INR sub-score					
0	474 (78.2)	2.1	1 [reference]	-	-
1	132 (21.8)	7.6	3.80	(1.53–9.47)	.005
FDP sub-score					
0	456 (75.2)	1.3	1 [reference]	-	-
1	88 (14.5)	5.7	4.52	(1.28–15.33)	.021
3	62 (10.2)	14.5	12.74	(4.42–39.33)	< 0.0001

*P* value <0.05.

OR, odds ratio; 95%CI, 95% confidence interval; JAAM-2, modified version of Japanese association for acute medicine; DIC, disseminated intravascular coagulation; PT-INR, prothrombin time-international normalized ratio; FDP, fibrin/fibrinogen degradation products.

**Figure 4:**
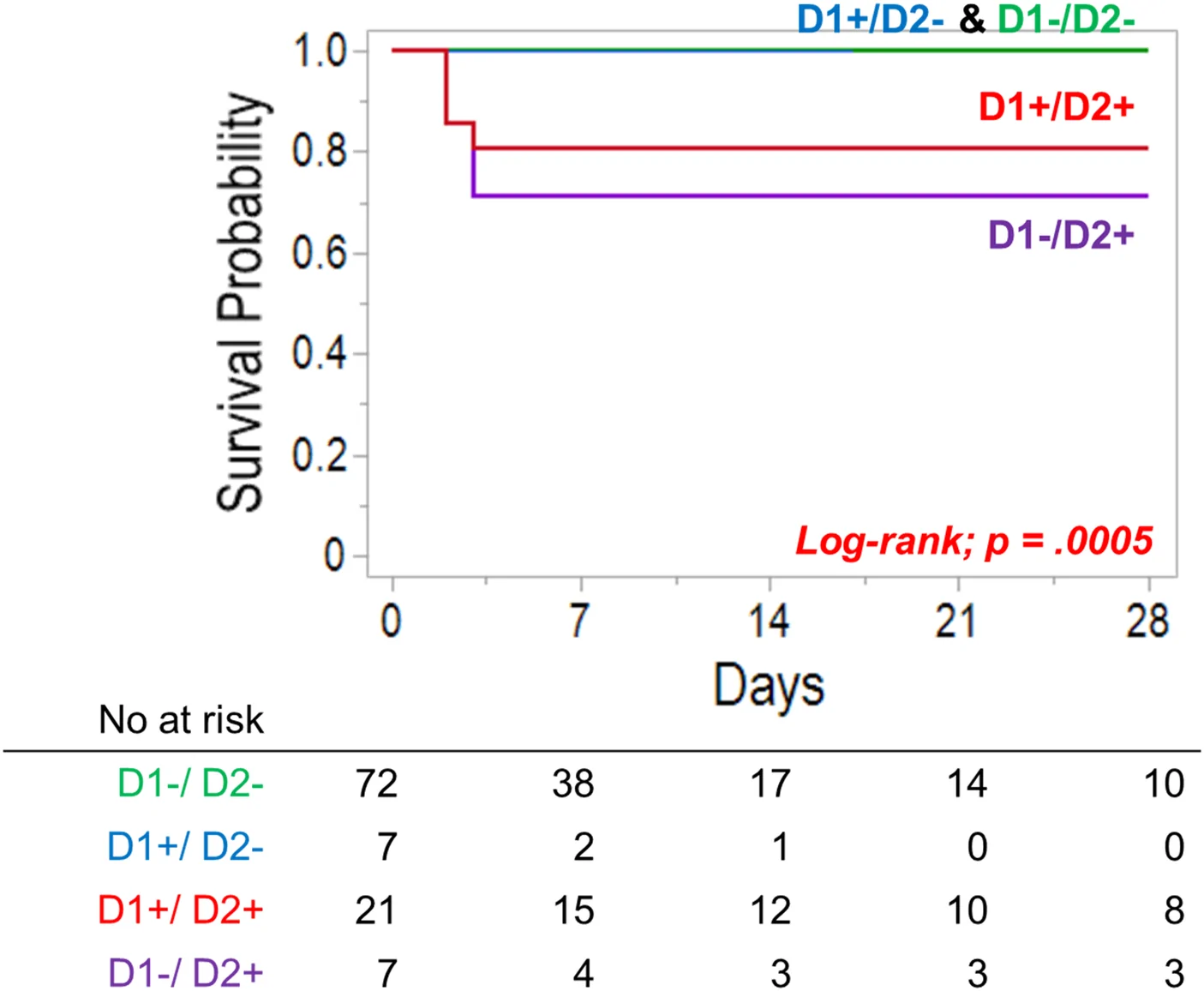
Survival analysis of each category of JAAM-2 DIC diagnosis within 2 days after heatstroke. In four categories, statistically significant differences in survival rates were observed. Specifically, the two groups diagnosed with DIC on the second day (“D1+/D2+” group and “D-/D+” group) had significantly worse outcomes. *D1* day1, *D2* day2.

A significant difference in survival probability was observed among the four DIC diagnostic categories (Log-rank test, *P* = 0.0005) (Figure 4). Specifically, all patients categorized as “D1-/D2-” (representing negative DIC on both Days 1 and 2) and “D1+/D2-” (representing positive DIC on Day 1 but negative on Day 2) all survived. In contrast, patients with D1-/D2+ (representing negative DIC on Day 1 but positive on Day 2) and D1+/D2+ (representing positive DIC on both Days 1 and 2) exhibited lower survival probabilities. No significant difference in survival probability was observed between the two groups that included deaths (Log-rank test; *p* = 0.6215) (Supplementary Figure 2).

## Discussion

This retrospective study demonstrated that DIC in patients with heatstroke is strongly correlated with poor clinical outcomes. Not only did patients meeting the JAAM-2 DIC criteria show higher mortality, but increases in sub-scores, particularly PT and FDP, were independently associated with elevated odds of in-hospital mortality. Furthermore, the presence of DIC on the second day after admission serves as a reliable risk factor of poor clinical outcomes.

An important feature of this study is that DIC was assessed using the JAAM-2 DIC score rather than the traditional JAAM DIC score. The JAAM-2 score was refined to exclude SIRS, making it more consistent with contemporary sepsis definitions and better aligned with pathophysiology-driven assessment (8). Although the JAAM-2 score has been validated in sepsis, evidence supporting its use for conditions other than infections remains limited. To our knowledge, this is the first study to evaluate the prognostic relevance of JAAM-2 DIC in heatstroke patients. Although the median JAAM-2 DIC score in the DIC group was close to the diagnostic threshold, the association between the JAAM-2 DIC score and mortality was graded across score levels and remained significant when the score was analyzed as a continuous variable. These findings suggest that the prognostic value of the JAAM-2 DIC score was not solely dependent on the dichotomous diagnostic threshold of ≥3.

Systematic inflammatory response is important in the pathophysiology of heatstroke (16). However, SIRS is present in the majority of heatstroke cases at the time of diagnosis, regardless of severity, reducing the clinical discriminatory capacity of SIRS-based scoring systems. Prior reports show SIRS positivity in up to 84% of exertional heatstroke cases on admission (17). Moreover, SIRS performs poorly in predicting mortality compared with organ dysfunction–based indices or DIC scoring (18). The present findings, therefore, support the need for diagnostic and prognostic tools independent of SIRS, so as to align with trends in sepsis research.

Consistent with earlier studies using the JAAM DIC score (3, 19), DIC remained a strong risk factor of mortality in heatstroke. In this predominantly elderly cohort, higher JAAM-2 DIC scores were associated with higher in-hospital mortality, particularly among patients with scores ≥4. This suggests that abnormal findings in multiple components of coagulation and fibrinolytic systems indicate progression to clinically significant DIC. In addition, among components of the JAAM-2 DIC score, platelet count and PT-INR were significantly lower and prolonged in the DIC group, although median values remained within the normal range. In contrast, FDP levels were markedly elevated (median 37.4 μL/mL), indicating substantial activation of the fibrinolytic pathway. These findings suggest that DIC associated with heatstroke may initially be characterized by systemic inflammation, followed by secondary activation of fibrinolysis (2). A recent ICU-based study by Endo et al. reported similar findings and further demonstrated that platelet coagulation fractions assessed via viscoelastic testing correlated with the severity of heatstroke-related DIC (20). The absence of an association between platelet count and mortality in this study suggests that platelet dysfunction, rather than thrombocytopenia, may be more strongly linked to prognosis.

This study also highlights the importance of monitoring DIC during the first two days after hospital admission. Patients who are positive for DIC on Day 2 had significantly poorer outcomes, suggesting that early reversal of coagulopathy may be crucial. Comparison of these four groups revealed that the two groups showing mortality (D1+/D2+, D1-/D2+) included a high proportion of severe heatstroke cases (Supplementary Table 5). In particular, the D1-/D2 + group had the lowest median age, but required mechanical ventilation, renal replacement therapy, vasopressors, anti-DIC therapy, and blood transfusions in many cases, suggesting severe organ failure. For heatstroke-related DIC, which resembles septic DIC, characterized by a suppressed-fibrinolysis phenotype (2), anticoagulant therapy is expected to improve clinical outcomes (4). Future prospective trials are needed to determine whether targeted DIC treatment can improve outcomes in this population.

There are some limitations in the present study. First, the sample size was not large. Second, due to the nature of this database, the reasons for measuring the DIC score and coagulation-fibrinolytic markers were unclear, and the possibility of selection bias cannot be ignored. Third, vital signs, including body temperature, could not be obtained, making it impossible to assess chronological changes in body temperature. Fourth, specific D-dimer assay kits were unidentifiable in the JMDC database. Although we applied validated fixed cut-off values (6 and 15 μg/mL) based on a recent cohort study (14), this lack of kit-specific calibration may have introduced misclassification bias regarding DIC severity. Fifth, data regarding exertional activity, detailed baseline comorbidities, cooling strategies, initial fluid volume, and neurologic progression were unavailable, limiting the assessment of patient heterogeneity, residual confounding, and treatment effects, and precluding reliable differentiation between classic and exertional heatstroke. Sixth, although treatment-related information, including the use of recombinant thrombomodulin, antithrombin, and heparin, was available, the exact timing of treatment initiation, dosage, duration, and interval between treatment administration and laboratory assessment were unavailable. Therefore, the potential influence of DIC-directed therapies on subsequent DIC scores, particularly the Day 1-to-Day 2 DIC transition, could not be fully evaluated. Finally, because the study population was predominantly elderly, the findings may not be generalizable to younger patients with exertional heatstroke or to populations with different baseline comorbidity profiles.

## Conclusions

In this retrospective analysis of a national Japanese medical claims database, JAAM-2 DIC was associated with poor clinical outcomes in patients with heatstroke. These findings highlight the clinical relevance of coagulopathy and suggest that DIC status during the first two days after admission may be associated with patient prognosis.

## Data Availability

The original contributions presented in the study are included in the article/Supplementary Material, further inquiries can be directed to the corresponding author.
