# Supplementary material for: Disseminated intravascular coagulation as a prognostic factor in heatstroke: a retrospective cohort study based on Japanese insurance claims
Source: Front Med (Lausanne). 2026 Sep 4;13:1941386. doi: 10.3389/fmed.2026.1941386 (PMC13585736; doi:10.3389/fmed.2026.1941386)
Supplement: Supplementary file 1 [file Datasheet1.docx]

**Supplementary Materials**

**Disseminated Intravascular Coagulation as a Prognostic Factor in Heatstroke: A Retrospective Cohort Study Based on Japanese Insurance Claims**

Hiroyuki Koami^1,2^*, Yutaro Furukawa^1,2^, Hiroki Ushijima^1,2^, Daisuke Muroya^2,3^, Tadashi Matsuoka^2,4^, Takaaki Totoki^2,5^, Kenji Kandori^2,6^, Takahiro Oishi^2,7^, Kasumi Satoh^8^, Yutaka Umemura^2,9^, Kazuma Yamakawa^2,5^, Mineji Hayakawa^2,10^, Kohji Okamoto^2,11^, Yuichiro Sakamoto^1,2^

Affiliations

^1^Department of Emergency and Critical Care Medicine, Saga University, Saga, Japan

^2^LOCOMOCO (Landmark Of Clinical Observations in MicrOcirculation and Coagulation Outcomes) study group, Tokyo, Japan

^3^Department of Emergency Medicine, Kitakyushu City Yahata Hospital, Kitakyushu, Fukuoka, Japan

^4^Department of Emergency and Critical Care Medicine, Keio University, Tokyo, Japan

^5^Department of Emergency and Critical Care Medicine, Osaka Medical and Pharmaceutical University, Takatsuki, Osaka, Japan

^6^Department of Emergency and Critical Care Medicine, Japanese Red Cross Society Kyoto Daini Hospital Kyoto, Japan

^7^Department of Emergency and Critical Care Medicine, Kansai Medical University, Osaka, Japan

^8^Emergency and Critical Care Medicine Department, Akita University Graduate School of Medicine, Akita, Japan

^9^Department of Traumatology and Acute Critical Medicine, Osaka University Graduate School of Medicine, Suita, Japan

^10^Department Emergency and General Medicine, Sapporo City General Hospital, Sapporo, Japan

^11^Department of Surgery, Kitakyushu City Yahata Hospital, Kitakyushu, Fukuoka, Japan

***Correspondence:**

Hiroyuki Koami

hkoami@cc.saga-u.ac.jp

Full postal address: 5-1-1 Nabeshima, Saga city, Saga, 849-8501, Japan

**Table S1. ICD-10 Coding Lists.**

| **Group** | **Code type** | **Code** |
| --- | --- | --- |
| **Bleeding Complications** | ICD-10 codes in the complications arising after admission | *Intracranial hemorrhages* I60.x, I61.x, I62.x, S06.4–S06.6  *Gastrointestinal bleedings* I85.0, I86.4, K25.0, K25.2, K25.4, K25.6, K26.0, K26.2, K26.4, K26.6, K27.0, K27.2, K27.4, K27.6, K28.0, K28.2, K28.4, K28.6, K62.5, K92.0–K92.2  *Respiratory tract bleedings* J94.2, R04  *Renal and urinary tract bleedings* R31  *Ocular bleedings* H31.3, H35.6, H43.1, H45.0  *Retroperitoneal bleeding* K66.1  *Pericardial bleeding* I31.2  *Bleeding-related anemia* D50.0, D62 |
| **DVT/PE** | ICD-10 codes in the complications arising after admission | *DVT* I80.1-I80.3, I80.8-I80.9, I82.8-I82.9, O22.3, O22.9, O87.1  *PE* I26.0, I26.9 |
| **Heatstroke** | ICD-10 codes in the primary diagnosis or admission-precipitating diagnosis | T67.0-T67.9 |
| *ICD-10* indicates International Classification of Diseases-Tenth Revision, *DVT* deep vein thrombosis, *PE* pulmonary embolism | | |

**Table S2. Definitions.**

| **Terms** | **Definitions** |
| --- | --- |
| **Severe grade of Heatstroke by JAAM-HS criteria** | ICD-10 code: T67.0  And any of the following conditions below:  1. Central nervous system manifestation (impaired consciousness based on the Japan  Coma Scale 2 or higher)  2. Hepatic/renal dysfunction required in-hospital care (T-BiL ≥ 2.0 mg/dL/ Cre ≥  2.0 mg/dL)  3. DIC (ICD-10: D65) |
| **Japana Coma Scale** | **0 (Alert)** |
|  | 0. Fully alert |
|  | **1-digit (Awake without stimulation)** |
|  | 1. Awake but slightly confused |
|  | 2. Awake with impaired orientation |
|  | 3. Awake but cannot tell own name and birthday |
|  | **2-digit (Arousable with stimulation)** |
|  | 10. Opens eyes to verbal stimuli |
|  | 20. Arouses to loud voice or shaking |
|  | 30. Arouses only to repeated verbal and pain stimuli |
|  | **3-digit (Unarousable)** |
|  | 100. Pain response with purposeful movement |
|  | 200. Pain response with non-purposeful movement |
|  | 300. No response to any stimuli |

*JAAM-HS* Japanese association for acute medicine-heatstroke, *ICD-10* indicates International Classification of Diseases-Tenth Revision, *T-BiL* total bilirubin, *Cre* creatinine, *DIC* disseminated intravascular coagulation

**Table S3. JAAM-2 DIC and ISTH-overt DIC criteria.**

| **Variables** | **JAAM-2 DIC** | **ISTH-overt DIC** | **Point** |
| --- | --- | --- | --- |
| **Platelet counts** | < 80 x 10^9^/L or > 50% decrease within 24 hours |  | 3 |
|  |  | < 50 x 10^9^/L | 2 |
|  | ≥ 80, < 120 x 10^9^/L or > 30% decrease within 24 hours | ≥ 50, < 100 x 10^9^/L | 1 |
|  | ≥ 120 x 10^9^/L | ≥ 100 x 10^9^/L | 0 |
| **PT  (ratio or time)** |  | ≥ 6 seconds | 2 |
|  | ≥ 1.2 | ≥ 3, < 6 seconds | 1 |
|  | < 1.2 | < 3 seconds | 0 |
| **FDP** | ≥ 25 mg/L |  | 3 |
|  | ≥ 10, < 25 mg/L |  | 1 |
|  | < 10 mg/L |  | 0 |
| **Elevated**  **fibrin-related**  **marker** |  | Strong increase | 3 |
|  |  | Moderate increase | 2 |
|  |  | No increase | 0 |
| **Fibrinogen** |  | < 100 g/mL | 1 |
|  |  | ≥ 100 g/mL | 0 |
| **Diagnosis** | ≥ 3 | ≥ 5 |  |

*JAAM-2* Japanese association for acute medicine-2, *DIC* disseminated intravascular coagulation, ISTH International society on thrombosis and haemostasis, *PT* prothrombin time, *FDP* fibrin/fibrinogen degradation products

**Table S4. Sensitivity analysis of the association between JAAM-2 DIC score and in-hospital mortality after adjustment for baseline comorbidity burden.**

| Model | OR  (JAAM-2 DIC score) | 95%CI | P value |
| --- | --- | --- | --- |
| Age-adjusted (Original model) | 1.566 | 1.147-2.140 | 0.004 |
| Modified Charlson index-adjusted | 1.633 | 1.202-2.227 | 0.002 |
| Age + Modified Charlson index | 1.595 | 1.166-2.183 | 0.003 |

*p* <0.05 is considered significant.

*OR* odds ratio, *95%CI* 95% confidence interval, *JAAM-2* modified version of Japanese association for acute medicine, *DIC* disseminated intravascular coagulation.

**Table S5. Patient populations experiencing heatstroke with DIC.**

|  | D1+/D2+  (N = 21) | D1+/D2-  (N = 7) | D1-/D2+  (N = 7) | D1-/D2-  (N = 72) | p value |
| --- | --- | --- | --- | --- | --- |
| Age, y/o | 85 [75-89] | 88 [70-93] | 72 [49-75] | 75 [63-83] | .002 |
| Male, N (%) | 9 (42.9) | 5 (71.4) | 6 (85.7) | 50 (69.4) | .085 |
| JAAM-HS, severe, N (%) | 18 (85.7) | 4 (57.1) | 7 (100.0) | 44 (61.1) | .040 |
| JCS on admission, N (%) |  |  |  |  |  |
| 0 | 7 (33.3) | 1 (14.3) | 1 (14.3) | 31 (43.1) | .013 |
| 1-3 | 6 (28.6) | 4 (57.1) | 0 (0.0) | 24 (33.3) |  |
| 10-30 | 3 (14.3) | 2 (28.6) | 1 (14.3) | 5 (6.9) |  |
| 100-300 | 5 (23.8) | 0 (0.0) | 5 (71.4) | 12 (16.7) |  |
| Platelet, 10^4^/µL | 12.4 [9.3-19.0] | 17.7 [17.4-23.4] | 13.2 [10-16.6] | 20.6 [16.1-26.7] | <.0001 |
| T-BiL, mg/dL | 1.1 [0.6-1.6] | 0.8 [0.5-1.4] | 0.7 [0.5-2.5] | 0.9 [0.6-1.2] | .457 |
| Cre, mg/dL | 1.4 [0.9-2.7] | 1.4 [0.9-2.2] | 1.5 [1.2-1.7] | 1.4 [0.9-1.92] | .955 |
| Mechanical ventilation, N (%) | 3 (14.3) | 0 (0.0) | 3 (42.9) | 2 (2.8) | .0007 |
| Renal replacement therapy, N (%) | 0 (0.0) | 0 (0.0) | 2 (28.6) | 0 (0.0) | <.0001 |
| Vasopressor, N (%) | 6 (28.6) | 0 (0.0) | 3 (42.9) | 1 (1.4) | <.0001 |
| AT, N (%) | 0 (0.0) | 0 (0.0) | 1 (14.3) | 0 (0.0) | .002 |
| rTM, N (%) | 2 (9.5) | 0 (0.0) | 2 (28.6) | 0 (0.0) | .0007 |
| Heparin, N (%) | 5 (23.8) | 1 (14.3) | 2 (28.6) | 5 (6.9) | .098 |
| Transfusion, N (%) | 5 (23.8) | 0 (0.0) | 3 (42.9) | 1 (1.4) | < .0001 |

Continuous values are expressed as medians [Q1, Q3]. Nominal variables are expressed as numbers (percent). *p* <0.05 is considered significant.

*JAAM-HS* Japanese association for acute medicine*, JCS* Japan coma scale*, T-BiL* total bilirubin*, Cre* creatinine*, AT* antithrombin*, rTM* recombinant thrombomodulin

**Figure S1. Study flow for secondary analysis.**

**Figure S2. Survival Analysis Comparing Category “D1+/D2+” and Category “D1-/D2+” of JAAM-2 DIC Diagnosis within 2 Days after Heatstroke.**
